# Personal

**Published:** 1912-06

**Authors:** 


### PERSONAL

Sir Bertram Dawson, Physician to King George V, recently passed through Buffalo and Niagara Falls, after visiting and studying with the Mayos of Rochester, Minn.

---

Dr. A. F. Miller of Batavia was thrown against a telegraph pole on May 3, something hving gone wrong with the steering gear of his automobile. He sustained no fractures but was badly shaken up.

---

The second congress of the Italian Alliance will be held in Buffalo, June 18-22. Drs. Charles R. Borzilleri, Joseph Tartaro and Anthony Cetola of Buffalo, are on the local committee.

---

Dr. Edmund E. Blaauw of Buffalo is spending the summer in Europe.

---

Dr. John G. Chadwick of Buffalo spent May in California.

Dr. Herman E. Hayd of Buffalo has been spending six weeks in California and other western states.

---

Dr. L. M. Wilkins of Lackawanna, had his automobile stolen while he was making a professionl call in Buffalo. The machine was later recovered at Black Rock but it had been driven hard and was badly damaged.

---

Dr. F. H. Sutterby of Bath visited in Leroy in May.

---

Dr. C. H. Thomas of Silver Springs has returned from Pine Bluff, N. C., where he has been spending the winter and spring.

---

Dr. George W. Pattison of Buffalo has moved to 84 Elmwood Ave.

---

Dr. Albert W. Palmer of Buffalo has moved to 1525 Seneca St.

---

Dr. DeLancey Rochester of Buffalo has moved his residence to 54 Ashland Ave., his office remaining at 469 Franklin St.

---

Dr. Charles Van Bergen of Buffalo, has returned from Paris and will be at home for a brief period.

---

Dr. Jacob E. Helwig of Martinsville has been re-elected School Commissioner, having held the office continuously for twenty years.

---

Dr. Charles E. Marshall, formerly of Fredonia, has been appointed Chief of the Dept. of Bacteriology at Amherst College.

---

Dr. Richard H. Satterlee of Buffalo has been appointed consulting ophthalmologist to the Thomas Indian School at Iroquois, N. Y.

---

Dr. Allen A. Jones of Buffalo has moved his residence to 487 Delaware Ave.

---

Dr. Frank C. Streeter has been elected President and Dr. C. F. Hoffman Health Officer of Bolivar, N. Y.

---

Dr. Walter S. Goodale of Buffalo, Supt. of the Ernest Wende Hospital and of the City Hospital for Contagious Diseases, will reside at the new cottage now being built on the grounds of the latter.

Dr. F. Parke Lewis of Buffalo addressed the graduates of the training school of the Memorial Hospital of Niagara Falls, May 14.

---

Drs. Earl P. Lothrop and Almon H. Cooke presented diplomas and gave brief addresses at the annual commencement of the training school of the Buffalo Woman's Hospital, May 16, 1912.

---

Dr. Grover W. Wende of Buffalo attended and presided at the annual meeting of the American Dermatologic Association in St. Louis, late in May.

---

Dr. David A. Gorton of Brooklyn, aged 80, is the father of twins, a boy and a girl. He is a vegetarian as well as an octogenarian.

---

Dr. S. A. Dunham of Buffalo, was a delegate to the Presbyterian convention at Louisville, in May.

---

Dr. Henry T. Williams and Dr. Charles R. Barber have both been appointed to the Park Board of the City of Rochester.

---

Dr. J. R. Williams of Rochester is in Europe.

---

Dr. Edward A. French, Rochester, has removed his office from Andrews Street to 209 Alexander Street, corner Monroe Avenue, being in the same house with his brother Dr. Robert T. French.

---

Dr. George G. Carroll, Rochester, has returned from a two years' residence in Vienna where he has been engaged in the study of eye, ear, nose and throat diseases.

---

Dr. Donald F. Macdonell has removed to 919 Hudson avenue, Rochester.

---

Dr. Peter C. Guinan, Rochester, has recovered from his recent illness.

---

Dr. George W. Goler, Dr. Edward Wheelock and Dr. C. R. Sumner are the physicians of Rochester mentioned in the new Who's Who in America for 1912.

